# The multifaceted regulatory effect of icariin on macrophages: a mini-review

**DOI:** 10.3389/fimmu.2026.1765533

**Published:** 2026-01-21

**Authors:** Juan Gao, Jin-Hong Gao, Yan-Fen Zhang, Da Gao, Ya-Peng Zhang

**Affiliations:** Department of Hematology, The Affiliated Hospital of Inner Mongolia Medical University, Hohhot, China

**Keywords:** bone-related diseases, cancer, icariin, inflammation, macrophages, targeted delivery system

## Abstract

Macrophages are the major type of innate immune cells. They play important regulatory roles in tissue homeostasis, immune defense, and pathological progression across diverse diseases. Icariin (ICA), a bioactive flavonol glycoside isolated from the genus *Epimedium*, exhibits extensive therapeutic potential by targeting macrophages in various conditions. This review summarizes the regulatory effect of ICA on macrophages and the underlying mechanisms. Specifically, ICA exerts a context-dependent regulatory effect on macrophage polarization, metabolic reprogramming, autophagy, and crosstalk with other cells. Furthermore, the potential of ICA as a therapeutic agent for treating inflammation, cancers, bone-related disorders, and fibrotic diseases is discussed. The discussion focuses on macrophage-dependent mechanisms. To address poor aqueous solubility and targetability of ICA, various delivery systems have been developed. These systems include exosomes, hydrogels, nanoparticles, scaffolds, and inhalable micelles. Their main purpose is to enhance the macrophage-specific accumulation of ICA. Overall, this review provides a comprehensive framework for understanding the modulatory effects of ICA on macrophages. It may offer beneficial references for future research and development of ICA as an immunomodulator. Specifically, it serves as a reference for developing ICA to treat macrophage-mediated diseases.

## 1 Introduction

Macrophages are evolutionarily conserved innate immune cells. They serve as critical regulators of tissue homeostasis, immune defense, and pathological progression across diverse diseases (1–3). Originating from BM-derived monocytes or tissue-resident progenitors, macrophages exhibit remarkable heterogeneity. They showed distinct names and functional specializations tailored to their tissue microenvironment (4). Macrophages are termed Kupffer cells in the liver responsible for clearing pathogens and metabolic waste while mediating hepatic inflammation in non-alcoholic steatohepatitis (5). In the central nervous system, microglia (the resident macrophage population) maintain synaptic pruning and neuroprotection. Yet, their dysactivation drives neuroinflammation in Alzheimer’s disease and multiple sclerosis (6). Macrophages interact with osteoblasts and osteoclasts to regulate bone remodeling in bone tissue (7). Tumor-associated macrophages (TAMs) in the tumor microenvironment (TME) promote tumor angiogenesis, immune suppression, and metastasis (8). This tissue-specific functional adaptation underscores macrophages as crucial therapeutic targets for diseases spanning inflammation, neoplasms, and musculoskeletal disorders. A defining feature of macrophages is their phenotypic plasticity, most prominently characterized by the dichotomous M1/M2 polarization paradigm. M1 macrophages, activated by lipopolysaccharide (LPS) or interferon-γ (IFN-γ), adopt a pro-inflammatory phenotype. They secrete pro-inflammatory cytokines, generate reactive oxygen species (ROS) via the nicotinamide adenine dinucleotide phosphate (NADPH) oxidase pathway. M1 macrophages also upregulate glycolysis for rapid energy production, which is critical for pathogen clearance but also contributing to tissue damage in chronic inflammation (9). In contrast, M2 macrophages, activated by interleukin-4 (IL-4) or IL-13, exhibit anti-inflammatory and tissue-reparative properties. They secrete IL-10 and transforming growth factor-β (TGF-β) to resolve inflammation, enhance arginase-1 (Arg-1) activity for tissue remodeling, and rely on oxidative phosphorylation for metabolism (9). However, M2 polarization is not universally beneficial, M2-like TAMs foster tumor immune evasion, while pro-fibrotic M2 macrophages drive extracellular matrix deposition in pulmonary or renal fibrosis (10, 11). This context-dependent functional duality of macrophages highlights the need for targeted regulators that can modulate polarization to restore homeostasis in specific pathological states. Dysregulated macrophage function is a common pathogenic thread in numerous diseases (1–3). Synovial M1 macrophages release matrix metalloproteinases (MMP) and pro-inflammatory cytokines in rheumatic arthritis (RA), leading to synovitis and cartilage erosion (12). Alveolar macrophages exhibit aberrant M1 polarization, triggering airway hyperresponsiveness and eosinophil infiltration in asthma (13). In atherosclerosis, macrophages accumulate oxidized low-density lipoprotein to form foam cells, while M1 polarization exacerbates plaque instability (14). Senescent macrophages (S-MΦs) and pro-osteoclastogenic macrophages disrupt the osteoblast-osteoclast balance, promoting bone resorption in bone-related disorders (15). TAMs suppress cytotoxic T cell activity via programmed death-ligand 1 (PD-L1) expression and secrete vascular endothelial growth factor (VEGF). This supports tumor angiogenesis and makes them key mediators of treatment resistance (16). Given these critical roles, modulating macrophage function has emerged as a promising therapeutic strategy.

Icariin (ICA) is a bioactive flavonol glycoside isolated from the genus *Epimedium* (commonly known as “Horny Goat Weed”) (17). Chemically, ICA has a molecular formula of C_33_H_40_O_15_ and a molecular weight of 676.66 g/mol (**Supplementary Figure 1**). Its structure is characterized by a flavonoid scaffold conjugated to sugar moieties. Such features that contribute to its biological activities yet limit its clinical utility due to poor aqueous solubility and low oral bioavailability (18). Preclinically, ICA has demonstrated diverse pharmacological effects, including anti-inflammatory, antioxidant, osteoprotective, immunomodulatory, and anti-tumor properties (19–22). While existing reviews have summarized the general immunomodulatory roles of ICA or its effect in specific diseases. Nevertheless, they lack a focused analysis of its interactions with macrophages, a critical gap given the growing body of evidence linking ICA’s therapeutic efficacy to macrophage regulation (23–25). In recent years, significant advancements have been made in this field. Novel mechanisms (e.g., ICA-mediated macrophage metabolic reprogramming, or autophagy activation in S-MΦs) have been identified, and macrophage-targeted delivery systems (e.g., adipose-derived stem cell exosomes, 3D-printed scaffolds) have been developed to enhance the efficacy of ICA (12, 15, 26). Additionally, the context-dependent regulation of macrophage polarization by ICA has been uncovered, highlighting its unique therapeutic potential (11, 12, 27). The present review focuses exclusively on the regulatory effects of ICA on macrophages and the underlying mechanisms. Furthermore, the therapeutic value of ICA in inflammation, bone disorders, cancer, and fibrosis are discussed, emphasizing macrophage-dependent mechanisms. This review aims to provide a comprehensive framework for understanding ICA’s macrophage-targeted potential and guiding future translational research.

## 2 Mechanisms of ICA in regulating macrophage function

### 2.1 Macrophage polarization regulated by ICA

**Figure 1:**
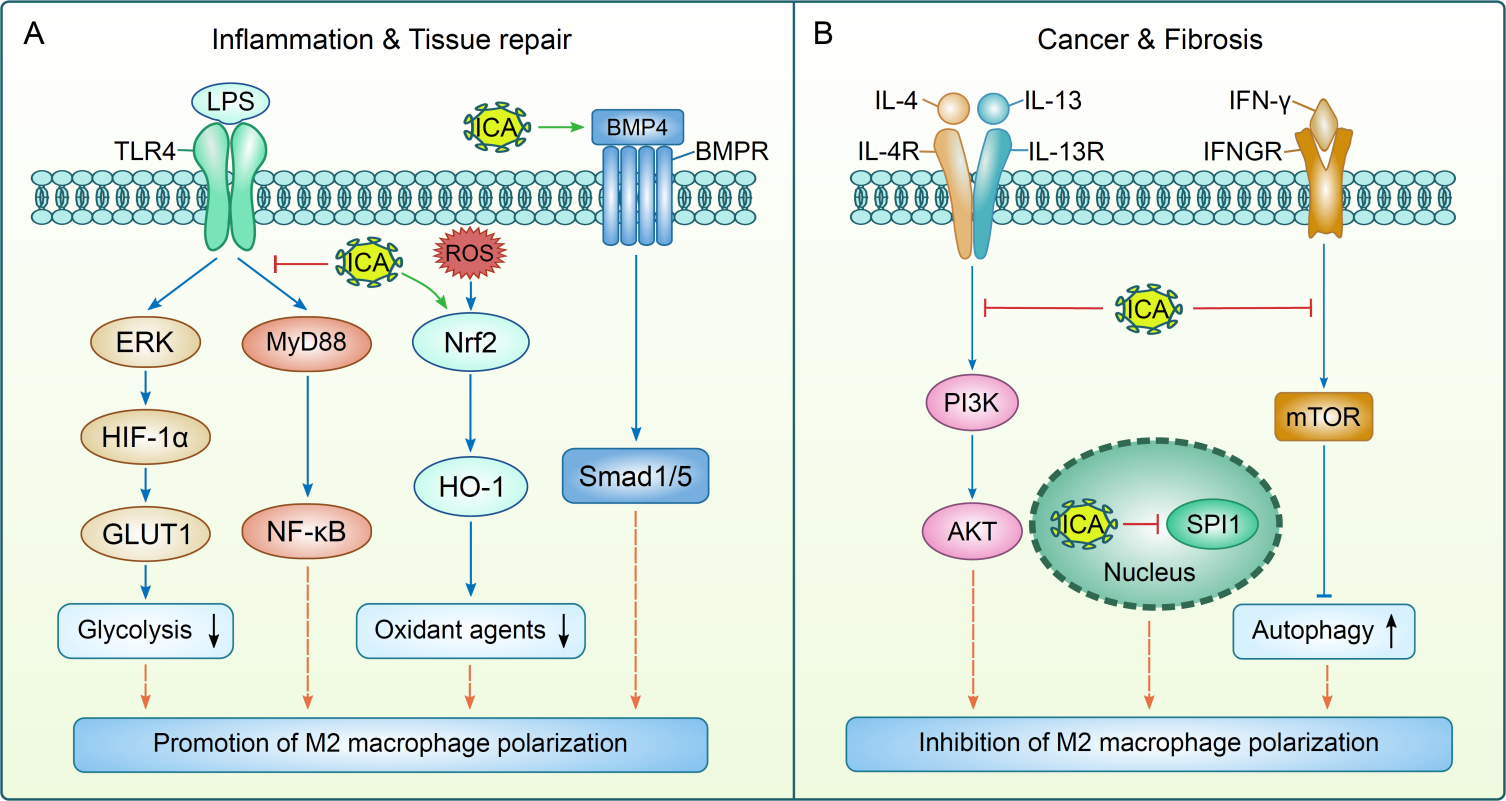
The mechanisms of icariin-mediated regulatory effect on macrophages. Icariin (ICA) exerts dual regulatory effects on M2 macrophage polarization to mediate distinct biological outcomes. In the contexts of inflammation resolution and tissue regeneration, ICA facilitates M2 polarization by inhibiting two key signaling cascades: the extracellular signal-regulated kinase (ERK)/hypoxia-inducible factor-1α (HIF-1α)/glucose transporter 1 (GLUT1) pathway (glycolysis suppression) and the toll-like receptor 4 (TLR4)/myeloid differentiation factor 88 (MyD88)/nuclear factor-κB (NF-κB) pathway. Moreover, ICA augments the nuclear factor erythroid 2-related factor 2 (Nrf2)/heme oxygenase-1 (HO-1)-driven antioxidative response and activates the bone morphogenetic protein 4 (BMP4)/mothers against decapentaplegic homolog 1/5 (Smad1/5) axis to further induce M2 macrophage differentiation. Conversely, in cancer and fibrotic pathologies, ICA restrains M2 macrophage polarization through targeted inhibition of the phosphatidylinositol 3-kinase (PI3K)/protein kinase B (AKT) pathway, mechanistic target of rapamycin (mTOR) (autophagy activation), and Spi-1 proto-oncogene (SPI1). LPS: lipopolysaccharide; BMPR: bone morphogenetic protein receptor; ROS: reactive oxygen species; IL-4R: IL-4 receptor; IL-13R: IL-13 receptor; IFN-γ: interferon-γ; IFNGR: interferon-γ receptor.

As a core functional characteristic of macrophages, polarization determines their functional orientation in various pathological and physiological processes. Therefore, macrophages are a key target for natural active compounds represented by ICA to exert regulatory effects. The regulatory effect of ICA on macrophage polarization shows highly context-dependent features. In inflammatory contexts, ICA suppresses M1 activation and promotes M1-to-M2 transition (**Supplementary Figure 2** and **Figure 1A**). It suppressed LPS + IFN-γ-induced M1 proliferation by reducing tumor necrosis factor-α (TNF-α)/IL-1β and upregulated M2 markers, Arg-1 and IL-10. The mechanisms included inhibition of extracellular signal-regulated kinase (ERK)/hypoxia-inducible factor-1α (HIF-1α)/glucose transporter 1 (GLUT1) and toll-like receptor 4 (TLR4)/myeloid differentiation factor 88 (MyD88)/nuclear factor-κB (NF-κB) (12, 27). ICA repressed alveolar macrophage M1 polarization by regulating a series of target genes in asthma models (13). In multiple sclerosis (MS), it mitigated cuprizone-induced demyelination by suppressing microglial M1 polarization via TLR4/nuclear factor-κB (NF-κB). Moreover, enhancement of nuclear factor erythroid derived 2 (Nrf2)/heme oxygenase-1 (HO-1)-mediated antioxidant effect was also involved (28). Similarly, the promotion of M2 polarization by ICA could also be observed in tissue repair progress. It activated bone morphogenetic protein 4 (BMP4)/mothers against decapentaplegic homolog 1/5 (Smad1/5), inducing M2 polarization to reduce inflammation and promote hair follicle neogenesis (29). In periodontitis, it drove M1-to-M2 transition and facilitated alveolar bone regeneration (30). Conversely, in fibrotic settings, ICA targeted pro-fibrotic M2-like macrophages (**Figure 1B**). The metabolite icariside II (ISE II) of ICA inhibited the infiltration of M2 macrophages (downregulating CD206/Arg-1 expression) via WNT/β-catenin suppression and reducing inflammatory factors (IL-1β, TNF-α) (10). An ICA-containing effective-compound combination (ECC) further inhibited M2 polarization via mechanistic target of rapamycin (mTOR) suppression (31). Likewise, in tumor microenvironments (TME), ICA could inhibit the generation of M2-like TAMs. It inhibited Spi-1 proto-oncogene (SPI1, also known as PU.1), reducing CC motif chemokine ligand 5 (CCL5) secretion and blocking CCL5/CCR5-mediated osteoclastogenesis in prostate cancers with bone metastasis (11). For colorectal cancer (CRC), ICA inhibited M2 polarization via phosphatidylinositol 3-kinase (PI3K)/protein kinase B (AKT), reducing CRC cell proliferation/migration in co-cultures and delaying tumor growth in azoxymethane (AOM)/dextran sodium sulfate (DSS)-induced mouse models (32). Therefore, ICA inhibits inflammation and accelerates repair via M2 promotion, while blocks fibrosis and resolves tumors through M2 suppression (**Figure 1**). However, the molecular basis underlying this contradictory regulatory effect remains unclear. Caution should be exercised when investigating and interpreting the mechanism of action of ICA on macrophages under distinct pathological microenvironments, such as hypoxic and inflammatory conditions. Specifically, hypoxia activates HIF-1α, a key mediator that drives M2 macrophage polarization (e.g., within the tumor microenvironment), whereas inflammation engages inflammatory signaling pathways to facilitate M1 macrophage polarization (e.g., during infectious diseases) (9, 33). Nevertheless, these two microenvironmental factors may interact synergistically to accelerate disease progression. For instance, inflammatory cytokines can promote the expression of HIF-1α, and conversely, HIF-1α upregulates the secretion of inflammatory cytokines, thereby amplifying the inflammatory response (34). Thus, it warrants further in-depth investigation to clarify the key molecules or microenvironmental cues that mediate this context-dependent selectivity.

### 2.2 Metabolic reprogramming of macrophages regulated by ICA

Macrophage polarization is closely coupled with metabolic reprogramming, which provides energy and material basis for their functional realization. Thus, the regulation of ICA on macrophages also involves the modulation of metabolic pathways (**Supplementary Figure 2**). Macrophages are metabolically plastic immune cells which undergo dynamic metabolic reprogramming to adapt to diverse microenvironmental factors and pathological roles in diseases (12, 35). A central metabolic hallmark of M1 macrophages is their dependence on glycolysis for energy production, even under normoxic conditions. ICA suppressed M1 proliferation and promoted M1-to-M2 phenotypic transition by suppressing glycolytic metabolism. The underlying mechanism was mediated by reducing the expression of GLUT1 (a key glucose transporter), thereby attenuating glucose uptake and glycolytic flux in M1 macrophages (12). This metabolic reprogramming not only diminished the secretion of pro-inflammatory cytokines, but also enhanced functions of M2 macrophages (12). Iron metabolism is also tightly regulated by ICA to affect macrophage function, and they played a central role in systemic iron homeostasis by storing and recycling iron. Dysregulated iron metabolism induced reactive oxygen species (ROS) production via the Fenton reaction, promoting osteoclast differentiation and bone loss (36). ICA prevented iron overload-induced bone loss in mice by reducing iron accumulation in the bone marrow (BM), inhibiting ROS production, and protecting osteoblasts from iron-induced apoptosis (35). Mechanistically, ICA regulated systemic iron metabolism by increasing hepcidin (a key hormone that controls iron absorption and release) expression through activation of signal transducer and activator of transcription 3 (STAT3) and Smad1/5/8 signaling pathways (37). This hepcidin-dependent regulation reduced iron availability to macrophages, suppressing their pro-osteoclastogenic function and ROS-mediated inflammatory responses (35, 37). Moreover, ICA was reported to inhibit osteoclastogenesis in ovariectomized (OVX) rats by reducing Cullin 3-mediated Nrf2 ubiquitination and degradation, enhancing Nrf2 nuclear translocation and HO-1 expression to scavenge ROS and suppress oxidative stress in macrophages (38). In conclusion, ICA emerges as a promising natural compound that regulates macrophage metabolic reprogramming to restore immune and tissue homeostasis. Future studies should be conducted to explore the crosstalk between different metabolic pathways mediated by ICA and their tissue specificity.

### 2.3 Macrophage autophagy regulated by ICA

In addition to polarization and metabolic remodeling, programmed cellular processes such as autophagy are also crucial for maintaining macrophage homeostasis and functional stability. It has become another important pathway through which ICA regulates macrophage function (**Supplementary Figure 2**). Autophagy is a homeostatic mechanism for cellular clearance, whose dysregulation contributes to the pathogenesis of age-related diseases, autoimmune disorders, and fibrosis (15, 31). ICA-mediated autophagy activation has been linked to anti-aging and anti-fibrotic effects. In the development of osteoporosis, S-MΦs in the BM secreted excessive senescence-associated secretory phenotype (SASP) factors, creating an inflamed microenvironment that impairs bone formation. ICA activated autophagy in S-MΦs, exerting a potent anti-inflammaging effect by reducing SASP expression and rejuvenating the osteogenic capacity of senescent BM mesenchymal stem cells (15). Transcriptomic analysis identified the TNF-α signaling pathway as a key mediator, with autophagy activation directly modulating TNF-α-driven inflammatory responses in S-MΦs, ultimately alleviating bone loss in osteoporotic mice (15). Similarly, in pulmonary fibrosis, an ECC containing ICA suppressed M2 polarization in IL-4-treated macrophages by promoting autophagy (31). Mechanistically, ECC inhibited the mTOR signaling pathway (a negative regulator of autophagy), and an autophagy inhibitor abrogated the anti-fibrotic effect of ECC, confirming autophagy as a central mediator (31). These studies highlight that ICA regulates macrophage function through modulation of autophagy.

### 2.4 Crosstalk between macrophages and other cells regulated by ICA

Macrophages act as central regulators in tissue microenvironments by engaging in intercellular crosstalk. The regulatory effect of ICA on macrophages is ultimately manifested through modulating their communication with surrounding cells (**Supplementary Figure 2**). The underlying mechanisms involve the secretion of cytokines and exosomes (39, 40). This paracrine regulation shapes the function of neighboring cells (12, 41). In inflammatory contexts like arthritis, ICA reduced the secretion of pro-inflammatory cytokines (TNF-α, IL-1β, IL-6) while increasing anti-inflammatory factors (IL-10), which mitigated synovitis mediated by inflammatory cells and preserved chondrocytes (12). For bone metabolism, ICA modulated macrophage cytokine secretion to rebalance osteoclast-osteoblast activity. In RAW264.7 cells and BM macrophages, it suppressed receptor activator of NF-κB ligand (RANKL)-induced macrophage secretion of pro-osteoclastogenic cytokines via inhibiting NF-κB/mitogen-activated protein kinase (MAPK) signaling. Additionally, it targeted the estrogen receptorα (ERα)/c-Src/RANK pathway to reduce osteoclast differentiation- promoting factors. Nevertheless, in bone tissue engineering scaffolds, ICA drove macrophages to secrete cytokines that enhance BM mesenchymal stem cell (BMSC) osteogenic differentiation and inhibit osteoclast activity (42–45). In prostate cancer (PC) bone metastasis, ICA inhibited the secretion of the chemokine CCL5 by TAMs, which not only blocked CCR5-mediated osteoclast differentiation (alleviating bone destruction) but also impaired cancer cell metastasis (11). Beyond soluble factors, ICA modulated macrophage/osteoclast-derived exosomes to regulate osteoblast function. In infected bone defects, ICA upregulated the microphthalmia-associated transcription factor (MITF)/Ras-related protein Rab27A pathway in osteoclasts to increase exosome release. These exosomes were enriched in miR-331-3p, which targeted fibroblast growth factor 23 (FGF23) in osteoblasts to reduce the Wnt inhibitor DKK1 and upregulate alkaline phosphatase (ALP) (41). Even in diabetic bone repair, ICA-loaded 3D scaffolds induced macrophages to secrete pro-angiogenic cytokines and exosomes with osteogenic miRNAs. They synergized to resolve chronic inflammation, promote vascularization, and enhance BMSC osteogenesis, achieving complete femoral defect repair in rats (46). Collectively, these findings highlight that ICA’s effect converge on modulating macrophage paracrine function to coordinate the function of target cells, underscoring macrophages as a central hub in ICA-mediated regulation of pathological and regenerative processes.

## 3 Therapeutic potential of ICA via regulating macrophages in various diseases

### 3.1 Inflammatory diseases

**Figure 2:**
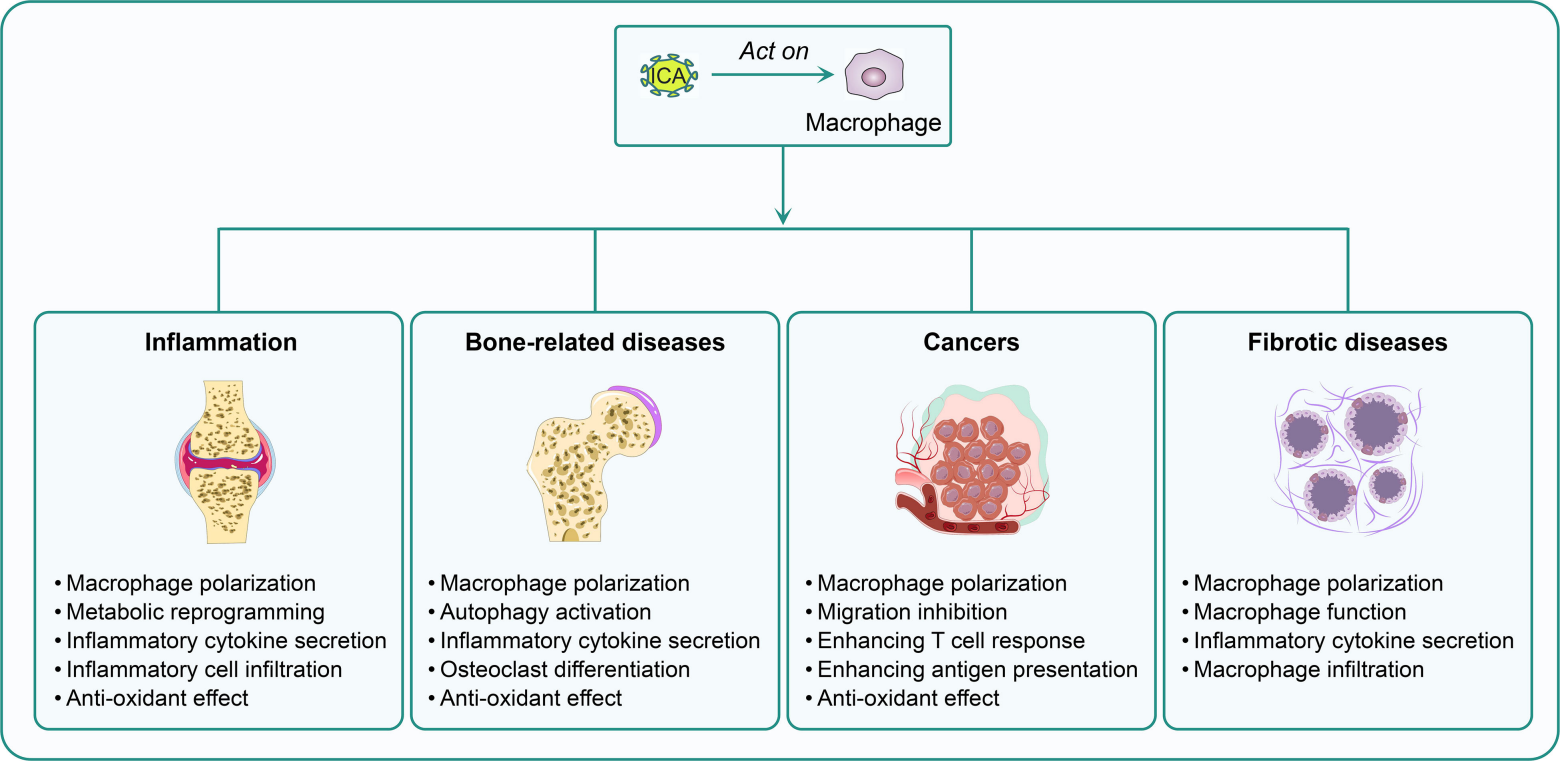
The therapeutic potential of ICA in different diseases. Icariin (ICA) is a potential agent for treating inflammatory, bone-related, and fibrotic diseases, as well as cancers. The underlying mechanisms include the modulation of macrophage polarization, metabolic reprogramming, inflammatory cytokine secretion, autophagy activation, and interactions with other immune cells, as described.

Macrophages play a central role in the initiation, progression, and resolution of various inflammatory diseases (47, 48). Many studies have indicated that ICA emerges as a promising therapeutic agent to treat inflammatory diseases via macrophage regulation (12, 13, 49–51). ICA exerted remarkable efficacy in rheumatoid arthritis (RA) and osteoarthritis by targeting synovial macrophages (**Figure 2**). For RA, it reduced cytokine levels, alleviated synovitis, and preserved cartilage in collagen-induced arthritis (CIA) rats (12). ICA could inhibit pro-inflammatory cytokines (IL-6, TNF-α) and upregulating anti-inflammatory IL-10 in LPS-activated macrophages. Intra-articular injection of ICA dose-dependently protects against cartilage degradation in monosodium iodoacetate (MIA)-induced osteoarthritis rats (49). Similarly, ICA suppressed macrophage-mediated inflammation and enhanced chondrocyte proliferation, supporting stable cartilage regeneration in a goat model (50). In respiratory inflammatory diseases like asthma and chronic obstructive pulmonary disease (COPD), ICA modulated alveolar macrophage function to ameliorate airway inflammation. In ovalbumin-induced asthmatic mice, ICA reduced M1 alveolar macrophage polarization, restored asthma-disrupted targets, and alleviated airway hyperresponsiveness and inflammatory cell infiltration, with its effects linked to metabolic reprogramming (13). For respiratory syncytial virus (RSV)-induced asthma, ICA ameliorated the sneezing and nose-scratching frequency and decreased OVA-specific IgE levels in mice. Moreover, it notably decreased the levels of inflammatory cytokine (IL-4, IL-5, IL-13) and infiltration of macrophages in the bronchoalveolar lavage fluid (BALF) (52). Jiang et al. developed a dry powder inhalation system for targeted pulmonary delivery of ICA, and found enhanced uptake of ICA-loaded micelles by RAW 264.7 macrophages and a 73% reduction in IL-4-induced CD206 expression. These results support the potential applicability of this inhalable system for modulating macrophage-mediated inflammation in COPD (51). Additionally, ICA as part of an ECC ameliorated the symptoms of COPD in rats by improving their lung function, reducing pathological changes, and suppressing oxidative responses and pro-inflammatory cytokine secretion (IL-1β, IL-6, IL-17, and TNF-α), while inhibiting inflammation in LPS-induced macrophages (53). In a relapse-remission experimental autoimmune encephalomyelitis (EAE) mouse model (SJL/J mice immunized with PLP139-151), ICA lowered clinical scores, reduced spinal cord microglial infiltration and demyelination, and downregulated inflammation-related signaling pathways, while suppressing pro-inflammatory markers (iNOS, TNF-α) (54). For systemic lupus erythematosus (SLE)-associated lupus nephritis (LN), ICA treatment reduced serum anti-dsDNA antibodies, immune complex deposition, and CCL2-mediated macrophage infiltration in MRL/lpr mice (55). ICA prevented macrophage-mediated lesion progression in atherosclerosis through two key mechanisms: downregulating CX3CR1 in macrophages to reduce arterial wall macrophage infiltration and lesion area in Apoe null mice (14). It also modulated scavenger receptors to suppress oxidized low-density lipoprotein (oxLDL)-mediated foam cell formation (56). Collectively, these studies demonstrate that ICA could affect diverse macrophage subtypes via condition-dependent mechanisms to treat distinct inflammatory diseases.

### 3.2 Bone-related diseases

Macrophages perform a crucial role in maintaining bone homeostasis by regulating the balance between osteoblast-mediated bone formation and osteoclast-driven bone resorption. Dysregulation of macrophage function contributes to the pathogenesis of various bone-related diseases, including osteoporosis, bone defects, periprosthetic osteolysis, and post-replantation root resorption (15, 35, 38, 57). ICA is a potential agent for bone disorders via macrophage regulation (**Figure 2**). For osteoporosis, regardless of the etiology (estrogen deficiency, iron overload, glucocorticoid exposure, or aging), ICA exhibited protective effects by suppressing macrophage-mediated inflammaging and osteoclast differentiation. In aged osteoporotic mice, ICA activated autophagy in S-MΦs, reducing their SASP and TNF-α signaling to rejuvenate osteogenic function of BMSC (15). For estrogen-deficient osteoporosis (ovariectomized), ICA inhibited RANKL-induced osteoclast differentiation from BM macrophages by targeting multiple pathways (35). It stabilized Nrf2 via inhibiting Cullin 3-mediated ubiquitination to reduce oxidative stress (38), and enhanced IGF-1-ERα crosstalk to promote osteogenesis (58). In iron overload-induced osteoporosis, ICA reduced macrophage-derived ROS production, protecting osteoblasts from apoptosis and inhibiting osteoclast activation (35). Similarly, in glucocorticoid-induced osteoporosis, ICA upregulated miR-186 to suppress cathepsin K expression in osteoclasts, reversing bone deterioration (57). For bone defect repair (including infected, traumatic, diabetic, and critical-sized defects), ICA modulated macrophage polarization to create a pro-regenerative microenvironment while balancing osteogenesis and osteoclastogenesis. Infected bone defects were ameliorated by ICA-enhanced osteoclast-derived exosomes and osteoblast activity was promoted (41). In diabetic bone defects, ICA induced M2 macrophage polarization, mitigating chronic inflammation and enhancing angiogenesis and osteogenesis (46). For critical-sized defects, ICA-loaded hydrogels and covalent organic framework nanoparticles achieved sustained ICA release, promoting M2 macrophage polarization and stimulating BMSC osteogenic differentiation (26, 45, 59). Additionally, ICA reduced root resorption after tooth replantation by inhibiting osteoclast differentiation from BM macrophages and human peripheral blood monocytes, while enhancing periodontal ligament stem cell osteogenesis (60). In periprosthetic osteolysis, a complication of total joint arthroplasty driven by wear particle-induced macrophage activation, ICA suppressed M1 macrophage polarization and pro-inflammatory cytokine (TNF-α, IL-6) production (61). It also reduced titanium particle-induced osteoclastogenesis, attenuated bone resorption and promoted bone formation (62, 63). Collectively, these studies demonstrate that ICA could restore bone homeostasis in various bone-related disease via modulating diverse macrophage populations.

### 3.3 Cancers

TAMs play a pivotal role in promoting tumor progression, metastasis, and immunosuppression (1). ICA and its derivative icaritin exert potent antitumor effects across multiple cancer types by targeting macrophage function (**Figure 2**), thereby disrupting pro-tumorigenic TME crosstalk and boosting anti-tumor immunity (32, 64). ICA inhibited the proliferation of cancer cells by suppressing macrophage-mediated pro-tumor signals. In CRC, a TME enriched with M2-polarized macrophages promoted CRC cell malignancy, and ICA reversed this by inhibiting M2 polarization of macrophages, as evidenced by decreased expression of M2 markers. In CRC cell-M2 macrophage co-cultures, ICA significantly suppressed CRC cell proliferation, migration, and invasion. In AOM/DSS-induced CRC and syngeneic CT26-WT implantation models, ICA attenuated tumor growth and reduced M2 macrophage infiltration (32). Similarly, in pancreatic cancer, ICA exerted dual effects. It directly inhibited Panc02 cell proliferation and migration while inducing apoptosis, and indirectly modulated the TME by suppressing M2 polarization of RAW264.7 cells. ICA also reduced infiltration of polymorphonuclear myeloid-derived suppressor cells, collectively inhibiting pancreatic tumor development (64). For tumor metastasis, ICA targeted macrophage-driven bone microenvironment remodeling in PC, a cancer with high bone metastasis rates. It suppressed PCa growth, bone metastasis, and osteoclastogenesis in a mouse PCa bone metastasis model. Mechanistically, ICA inhibited M2 polarization of TAMs derived from Raw264.7 cells, transcriptionally reducing their secretion of CCL5. Since CCL5 binds to its receptor CCR5 on osteoclast precursors to promote their differentiation and chemotaxis, ICA-mediated downregulation of the TAM/CCL5/CCR5 axis blocked PCa-induced bone destruction. This effect was validated by clinicopathological analysis showing a positive correlation between this axis and osteoclastogenesis in PCa patients (11). Moreover, ICA could enhance antitumor immunity. ICA and icaritin (ICA’s hydrolytic derivative) boost macrophage-dependent immune surveillance and T cell-mediated cytotoxicity. In a P815 mouse mastocytoma model, ICA acted as an antigen adjuvant. It combined with P815AB peptide to increase cytotoxic T lymphocyte (CTL) responses, elevate the percentage of activated T cells (CD4^+^CD8^+^, CD3^+^CD69^+^, CD69^+^NKG2D^+^), and enhance peritoneal macrophage function. Furthermore, ICA upregulated MHC-I-related molecules to improve tumor antigen presentation (65). Icaritin further synergized with immunomodulators in B16F10 melanoma models. Systemic icaritin plus intratumoral CpG reversed immunosuppression by increasing recruitment of functional dendritic cells (DCs) and TAMs, enhancing infiltration of cytotoxic CD8^+^ T cells, and augmenting the efficacy of anti-programmed death-1 (PD-1)/cytotoxic T-Lymphocyte-associated protein 4 (CTLA-4) checkpoint blockade (66). Notably, icaritin also showed promise in hepatocellular carcinoma (HCC), with clinical trials demonstrating improved survival in advanced HCC patients via modulating macrophage and other immune cell functions (67). Collectively, these findings demonstrate that ICA and its derivative icaritin modulate macrophages to exert multi-faceted antitumor effects. A key strength is their ability to act both directly on tumor cells and indirectly via TME modulation and crosstalk to other immune cells, making them effective across diverse cancers. Future research should focus on exploring its synergies with other immunotherapies (e.g., chimeric antigen receptor T cells). Such efforts could position ICA as a versatile adjuvant in cancer treatment, leveraging its ability to rewire the TME toward an anti-tumor state.

### 3.4 Fibrotic diseases

Fibrotic diseases, characterized by excessive extracellular matrix deposition, tissue remodeling, and progressive organ dysfunction, represent a major clinical challenge (68). In pulmonary fibrosis (PF), a leading fibrotic disorder, both ISE II and ICA-containing combinations could mitigate fibrosis via modulating macrophage polarization and function (**Figure 2**). ISE II, the key metabolite of ICA, alleviated bleomycin-induced PF in mice by improving lung function, reducing collagen deposition, and downregulating pro-fibrotic cytokines (IL-1β, TNF-α, TGF-β1) in serum and BALF (10). Similarly, an ECC containing ICA markedly suppressed macrophage infiltration and inhibited inflammatory responses, leading to reductions in lung injury. ECC treatment suppressed bleomycin-induced collagen deposition and collagen I, collagen III, and hydroxyproline levels (31). These findings demonstrate that ICA and its metabolites exert broad anti-fibrotic effect, and future research should explore the efficacy of ICA in other fibrotic models (e.g., hepatic, cardiac fibrosis).

### 3.5 Other diseases

Macrophages serve as pivotal regulators in immune defense and neuroprotection. In terms of antibacterial immunity, ICA rescued sepsis-induced immunosuppression by enhancing macrophage-mediated bacterial clearance. In a cecal ligation and puncture (CLP)-induced sepsis mouse model with secondary *Pseudomonas aeruginosa* infection, ICA significantly reduced organ damage and bacterial load. It also boosted phagocytosis and bactericidal capacity *in vitro* studies on endotoxin-tolerant BM-derived macrophages (BMDM). Mechanistically, ICA bound to ribosomal S6 Kinase 2 (RSK2), promoting Yes-associated Protein (YAP) phosphorylation and degradation to relieve YAP-mediated inhibition of cGAS, leading to enhancement of macrophage antibacterial activity and restoration of immune homeostasis (69). For neuroinflammation alleviation, ICA modulated macrophage/microglia polarization to suppress neurotoxicity. In a rat model of anterior ischemic optic neuropathy, ICA inhibited macrophage infiltration and optic nerve edema, preserved retinal ganglion cell density for one month, and activated the noncanonical NF-κB pathway via the CCAAT enhancer binding protein β (CEBP-β)/granulocyte colony-stimulating factor (G-CSF) axis. This drove M2 polarization of microglia/macrophages and AKT1 activation, preventing neuroinflammation and retinal ganglion cell apoptosis (70). Moreover, in neurodegenerative disease contexts, ICA inhibited the nucleotide-binding oligomerization domain-like receptor family pyrin domain-containing 3 (NLRP3) inflammasome in microglia, reducing pro-inflammatory cytokine release and microglial neurotoxicity (71). Collectively, these findings indicate that ICA modulates macrophage function in infection immunity and neuroprotection.

## 4 Drug delivery systems for targeting macrophages with ICA

Although ICA is a promising agent for treating various disorders through orchestrating macrophage polarization and function, its clinical translation is significantly hindered by poor aqueous solubility, low permeability, and limited bioavailability. To address these limitations, various targeted delivery systems have been developed to improve its delivery efficiency aiming to enhance the interaction between ICA and macrophages (**Supplementary Table 1**). These systems are designed to leverage distinct carrier properties, either through passive accumulation at inflammatory sites or active interaction with macrophage-specific characteristics.

### 4.1 Exosome-based delivery systems

Exosomes derived from stem cells exhibit inherent biocompatibility, low immunogenicity, and natural tropism for inflammatory tissues, making them ideal carriers for macrophage targeting. Adipose-derived stem cell exosomes (ADSCs-EXO) loaded with ICA achieved high loading efficiencies (92.4 ± 0.008% and 92.7 ± 0.010%) (12, 27). These exosomes actively targeted macrophages in synovial tissue or inflammatory foci, facilitating efficient ICA delivery to the intended cell type. Notably, ADSCs-EXO-ICA demonstrated superior macrophage uptake compared to free ICA or exosome-only treatments, enabling effective modulation of macrophage behavior through sustained ICA release (27). These findings underscore the potential value of exosome-based delivery systems as a natural carrier for ICA delivery.

### 4.2 Hydrogel-based delivery systems

Hydrogels are widely used for local ICA delivery due to their injectability, biodegradability, and ability to achieve controlled sustained release. Thermosensitive PEG hydrogels (29), chitosan (CTS) hydrogels (50), and silk fibroin-based double-network hydrogels (45) are among the commonly employed formulations. These hydrogels encapsulated ICA to prevent rapid degradation, with sustained release profiles ranging from weeks to months (50). Their high biocompatibility and ability to conform to tissue defects allowed localized accumulation at macrophage-rich sites (e.g., wound beds, bone defects), enhancing the accessibility of ICA to target cells without systemic side effects. Moreover, gallic acid-modified hydroxybutyl chitosan hydrogels encapsulating ICA-loaded zinc-aluminum layered double hydroxide nanosheets (GA-HBC-LIC) enabled sequential delivery, further optimizing macrophage targeting at inflammatory sites (30). These results indicate hydrogels are promising systems for local ICA delivery.

### 4.3 Nanoparticle-based delivery systems

Nanoparticle carriers leverage their small size to facilitate macrophage phagocytosis, a key passive targeting mechanism. ICA-loaded tannic acid-functionalized nanodiamonds (ICA/TA-NDs) (49) and polydopamine-modified covalent organic framework nanoparticles (ICA@PCOFs) (59) exhibited excellent colloidal stability and high ICA encapsulation efficiency. These nanoparticles were efficiently internalized by RAW 264.7 macrophages *in vitro*, leveraging the cells’ inherent phagocytic capacity for targeted delivery. Copper-doped mesoporous bioactive glass nanoparticles (BGNPs) co-delivering ICA and copper ions also enhanced macrophage uptake, with the nanoparticle structure enabling controlled ICA release (72). Thus, nanoparticles are potential carriers for loading ICA to target macrophages in different settings.

### 4.4 Scaffold-based delivery systems

3D-printed and surface-modified scaffolds are designed for localized, long-term ICA delivery in tissue regeneration contexts, targeting macrophages at defect sites. 3D-printed potassium sodium niobate/nano-hydroxyapatite/polylactic acid (KNN/nHA/PLA) scaffolds integrated with ICA-loaded GelMa hydrogel (26), poly(lactide-co-glycolide)/hydroxyapatite (PLGA/HA) scaffolds decorated with small intestine submucosa (46), and sulfonated polyetheretherketone (SPEEK) scaffolds modified with polydopamine (PDA)-ICA (73) provided mechanical support while enabling sustained ICA release. TiO2 nanotube (TNT) scaffolds functionalized with PDA-ICA (74) and hydroxyapatite/alginate (HAA) porous scaffolds (44) also enhanced localized ICA concentration at macrophage-rich implant sites, promoting targeted delivery through surface adsorption and controlled release.

### 4.5 Micelle-in-microparticle delivery systems

For pulmonary macrophage targeting, trehalose-stabilized DSPE-PEG2000/DPPC micelle-in-microparticles have been developed as powder inhalants (51). These formulations exhibited favorable aerodynamic properties (mass median aerodynamic diameter: 2.36 ± 0.3 µm; fine particle fraction: 44%), enabling efficient deposition in the lower respiratory tract and targeted uptake by alveolar macrophages. The micellar structure preserved ICA in an amorphous state, enhancing its solubility and macrophage internalization (51). Additionally, PLGA@ICA microspheres encapsulated in biomimetic scaffolds achieved high encapsulation efficiency and sustained release, further improving macrophage targeting at bone defect sites (75). Therefore, these results suggest that micelle-in-microparticles are another useful delivery system for local ICA delivery to regulate macrophages.

## 5 Summary and future perspectives

ICA displays broad therapeutic potential in a series of diseases by targeting macrophage function. The underlying mechanisms encompass regulating polarization, metabolic reprogramming, and autophagy of macrophages. To address the poor aqueous solubility and low bioavailability of ICA, diverse macrophage-targeted delivery systems have been developed. These systems include exosomes, hydrogels, nanoparticles, 3D scaffolds, and micelle-in-microparticles, significantly enhancing its targeted accumulation and efficacy. Substantial hurdles remain to fully exploit the therapeutic potential of ICA. The precise molecular mechanisms governing ICA’s regulation of tissue-specific macrophage subsets (e.g., TAMs, microglia, and synovial macrophages) *in vivo* are not fully elucidated. There is a lack of understanding regarding how ICA discriminates between functionally overlapping subtypes (e.g., pro-repair M2 macrophages versus pro-fibrotic M2-like macrophages) and the upstream signaling molecules or transcription factors that mediate this subtype specificity, with few studies leveraging *in vivo* dynamic tracking to capture real-time regulatory processes. Currently, no studies have evaluated the impact of ICA on macrophages in clinical patients. Only one clinical trial has investigated the anti-cancer effect of icaritin in patients with hepatocellular carcinoma. The results showed that icaritin treatment was associated with a higher level of PD-L1^+^ macrophages and a tendency toward superior overall survival (76). Clinical translation is impeded by the lack of direct clinical verification of macrophage-modulating effect of ICA in patients, its poor aqueous solubility and low bioavailability (which may compromise clinical efficacy), and the lack of standardized macrophage function biomarkers for designing trials in macrophage-mediated diseases. Additionally, macrophage-targeted delivery systems require further refinement. Some carriers may trigger unintended immune responses, while others suffer from mismatched degradation rates and drug release kinetics. Scalability for industrial production is another issue, as many advanced delivery systems are currently limited to laboratory-scale synthesis, with poor batch-to-batch consistency. Future research should strategically address these challenges to advance the clinical utility of ICA. Mechanistic investigations should integrate cutting-edge technologies such as single-cell RNA sequencing, spatial transcriptomics, and lineage tracing to dissect the subtype-specific regulatory effects of ICA on tissue-resident macrophages. This approach will help map disease-macrophage subtype-ICA mechanism relationships, and identify key molecular targets that drive subtype specificity. In combination therapy, exploring synergistic regimens tailored to disease types holds great promise. The combination of ICA with immune checkpoint inhibitors (e.g., anti-PD-1/CTLA-4) to rewire TAMs and enhance cytotoxic T cell infiltration in cancer, or pairing ICA with anti-osteoporotic drugs to synergistically inhibit osteoclastogenesis by regulating macrophages. Finally, optimizing the structure-activity relationship (SAR) of ICA through molecular docking and virtual screening is needed. Modification of its flavonoid scaffold to increase binding affinity to macrophage-specific receptors or introduction of hydrophilic moieties to improve solubility, will boost its macrophage-regulatory potency and bioavailability. Thus, ICA is promising agent for treating numerous disorders via regulating macrophages, although further studies are needed to optimize its utility and discover the precise mechanisms.

## References

[1] Mantovani A Marchesi F Di Mitri D Garlanda C . Macrophage diversity in cancer dissemination and metastasis. Cell Mol Immunol. (2024) 21:1201–14. doi: 10.1038/s41423-024-01216-z, PMID: 39402303 PMC11528009

[2] Cutolo M Soldano S Smith V Gotelli E Hysa E . Dynamic macrophage phenotypes in autoimmune and inflammatory rheumatic diseases. Nat Rev Rheumatol. (2025) 21:546–65. doi: 10.1038/s41584-025-01279-w, PMID: 40721670

[3] Kopecky BJ Lavine KJ . Cardiac macrophage metabolism in health and disease. Trends Endocrinol metabolism: TEM. (2024) 35:249–62. doi: 10.1016/j.tem.2023.10.011, PMID: 37993313 PMC10949041

[4] Peng M Li N Wang H Li Y Liu H Luo Y . Macrophages: subtypes, distribution, polarization, immunomodulatory functions, and therapeutics. MedComm. (2025) 6:e70304. doi: 10.1002/mco2.70304, PMID: 40717900 PMC12290311

[5] Nusse Y Kubes P . Liver macrophages: development, dynamics, and functions. Cell Mol Immunol. (2025) 22:1178–89. doi: 10.1038/s41423-025-01298-3, PMID: 40461700 PMC12480653

[6] Gao C Jiang J Tan Y Chen S . Microglia in neurodegenerative diseases: mechanism and potential therapeutic targets. Signal transduction targeted Ther. (2023) 8:359. doi: 10.1038/s41392-023-01588-0, PMID: 37735487 PMC10514343

[7] Weivoda MM Bradley EW . Macrophages and bone remodeling. J Bone mineral research: Off J Am Soc Bone Mineral Res. (2023) 38:359–69. doi: 10.1002/jbmr.4773, PMID: 36651575 PMC10023335

[8] Chu X Tian Y Lv C . Decoding the spatiotemporal heterogeneity of tumor-associated macrophages. Mol cancer. (2024) 23:150. doi: 10.1186/s12943-024-02064-1, PMID: 39068459 PMC11282869

[9] Bai X Guo YR Zhao ZM Li XY Dai DQ Zhang JK . Macrophage polarization in cancer and beyond: from inflammatory signaling pathways to potential therapeutic strategies. Cancer letters. (2025) 625:217772. doi: 10.1016/j.canlet.2025.217772, PMID: 40324582

[10] Deng L Ouyang B Shi H Yang F Li S Xie C . Icariside II attenuates bleomycin-induced pulmonary fibrosis by modulating macrophage polarization. J ethnopharmacol. (2023) 317:116810. doi: 10.1016/j.jep.2023.116810, PMID: 37331450

[11] Chen C Wang S Wang N Zheng Y Zhou J Hong M . Icariin inhibits prostate cancer bone metastasis and destruction via suppressing TAM/CCL5-mediated osteoclastogenesis. Phytomedicine: Int J phytotherapy phytopharmacology. (2023) 120:155076. doi: 10.1016/j.phymed.2023.155076, PMID: 37716031

[12] Yan Q Liu H Sun S Yang Y Fan D Yang Y . Adipose-derived stem cell exosomes loaded with icariin alleviates rheumatoid arthritis by modulating macrophage polarization in rats. J nanobiotechnol. (2024) 22:423. doi: 10.1186/s12951-024-02711-1, PMID: 39026367 PMC11256651

[13] Zhu X Wang B Yu H Li C Zhao Y Zhong Y . Icariin attenuates asthmatic airway inflammation via modulating alveolar macrophage activation based on network pharmacology and *in vivo* experiments. J Gene Med. (2024) 26:e3718. doi: 10.1002/jgm.3718, PMID: 38979822

[14] Wang Y Wang YS Song SL Liang H Ji AG . Icariin inhibits atherosclerosis progress in Apoe null mice by downregulating CX3CR1 in macrophage. Biochem Biophys Res Commun. (2016) 470:845–50. doi: 10.1016/j.bbrc.2016.01.118, PMID: 26802470

[15] Bai L Liu Y Zhang X Chen P Hang R Xiao Y . Osteoporosis remission via an anti-inflammaging effect by icariin activated autophagy. Biomaterials. (2023) 297:122125. doi: 10.1016/j.biomaterials.2023.122125, PMID: 37058900

[16] Wang H Yung MMH Ngan HYS Chan KKL Chan DW . The impact of the tumor microenvironment on macrophage polarization in cancer metastatic progression. Int J Mol Sci. (2021) 22:6560. doi: 10.3390/ijms22126560, PMID: 34207286 PMC8235734

[17] Cai Z Sun F Wang Q Li S Wang L Li H . Icariin alleviates cardiomyocyte pyroptosis through AMPK-NLRP3 pathway to ameliorates diabetic cardiomyopathy. Int immunopharmacol. (2025) 156:114690. doi: 10.1016/j.intimp.2025.114690, PMID: 40262250

[18] Jiang C Bai R Somavarapu S . Inhalable TPGS/DPPC micelles coloaded with curcumin and icariin for targeted lung cancer therapy. ACS omega. (2025) 10:15400–11. doi: 10.1021/acsomega.5c00008, PMID: 40290948 PMC12019740

[19] Zhai T Wang J Chen Y Su J Feng W . Icariin induces chondrocyte degeneration via activation of the NF-κB signalling pathway and reduces the inflammation factor expression induced by lipopolysaccharide. Immunology. (2025) 175:76–83. doi: 10.1111/imm.13906, PMID: 39949189

[20] Su L Wei ZF Pi CC Qin TX Song F Zhang YW . Icariin protects against acute graft-versus-host disease while preserving graft-versus-leukemia activity after allogeneic hematopoietic stem cell transplantation. Phytomedicine: Int J phytotherapy phytopharmacology. (2024) 132:155901. doi: 10.1016/j.phymed.2024.155901, PMID: 39067193

[21] Zhang Z Zhuang H Fang C Lai Y Zhang S Wang M . Icariin attenuates diabetic cardiomyopathy by activating Nrf2-dependent antioxidant and mitochondrial pathways: Integrative evidence from network pharmacology and experimental validation. J ethnopharmacol. (2026) 355:120667. doi: 10.1016/j.jep.2025.120667, PMID: 41038331

[22] Haoyue W Kexiang S Shan TW Jiamin G Luyun Y Junkai W . Icariin promoted ferroptosis by activating mitochondrial dysfunction to inhibit colorectal cancer and synergistically enhanced the efficacy of PD-1 inhibitors. Phytomedicine: Int J phytotherapy phytopharmacology. (2025) 136:156224. doi: 10.1016/j.phymed.2024.156224, PMID: 39642461

[23] Bi Z Zhang W Yan X . Anti-inflammatory and immunoregulatory effects of icariin and icaritin. Biomedicine pharmacotherapy = Biomedecine pharmacotherapie. (2022) 151:113180. doi: 10.1016/j.biopha.2022.113180, PMID: 35676785

[24] Shen R Wang JH . The effect of icariin on immunity and its potential application. Am J Clin Exp Immunol. (2018) 7:50–6., PMID: 30038846 PMC6055068

[25] Liu X Wang Z Qian H Tao W Zhang Y Hu C . Natural medicines of targeted rheumatoid arthritis and its action mechanism. Front Immunol. (2022) 13:945129. doi: 10.3389/fimmu.2022.945129, PMID: 35979373 PMC9376257

[26] Wang Y Zhang H Xu Z Zhu W Chang S Wei J . Icariin-loaded GelMa hydrogel encapsulated potassium sodium niobate biomimetic piezoelectric scaffold regulates macrophage polarization to accelerate bone defect repair. Materials Today Bio. (2025) 35:102476. doi: 10.1016/j.mtbio.2025.102476, PMID: 41281663 PMC12639499

[27] Yan Q Song C Liu H Li Y Ma J Zhao Y . Adipose-derived stem cell exosomes loaded with icariin attenuated M1 polarization of macrophages via inhibiting the TLR4/Myd88/NF-κB signaling pathway. Int immunopharmacol. (2024) 137:112448. doi: 10.1016/j.intimp.2024.112448, PMID: 38870883

[28] Song LJ Han QX Ding ZB Liu K Zhang XX Guo MF . Icariin ameliorates the cuprizone-induced demyelination associated with antioxidation and anti-inflammation. Inflammopharmacology. (2024) 32:809–23. doi: 10.1007/s10787-023-01388-6, PMID: 38177566

[29] Teng YY Zou ML Liu SY Jia Y Zhang KW Yuan ZD . Dual-action icariin-containing thermosensitive hydrogel for wound macrophage polarization and hair-follicle neogenesis. Front bioengineering Biotechnol. (2022) 10:902894. doi: 10.3389/fbioe.2022.902894, PMID: 35832407 PMC9272914

[30] Chen J Guan X Chen L Zheng B Li F Fang C . Customized hydrogel system for the spatiotemporal sequential treatment of periodontitis propelled by ZEB1. Advanced Sci (Weinheim Baden-Wurttemberg Germany). (2025) 12:e2503338. doi: 10.1002/advs.202503338, PMID: 40184628 PMC12245124

[31] Zhao P Cai Z Tian Y Li J Li K Li M . Effective-compound combination inhibits the M2-like polarization of macrophages and attenuates the development of pulmonary fibrosis by increasing autophagy through mTOR signaling. Int Immunopharmacol. (2021) 101:108360. doi: 10.1016/j.intimp.2021.108360, PMID: 34801418

[32] Chen Y Qi Y Jiang Y Li Y Yang S Wang L . Icariin modulates the tumor microenvironment in colorectal cancer by targeting M2 macrophage polarization via PI3K/AKT pathway. Bioorganic medicinal Chem. (2025) 129:118317. doi: 10.1016/j.bmc.2025.118317, PMID: 40683071

[33] Ambade A Satishchandran A Saha B Gyongyosi B Lowe P Kodys K . Hepatocellular carcinoma is accelerated by NASH involving M2 macrophage polarization mediated by hif-1αinduced IL-10. Oncoimmunology. (2016) 5:e1221557. doi: 10.1080/2162402X.2016.1221557, PMID: 27853646 PMC5087291

[34] Li C Wang Y Li Y Yu Q Jin X Wang X . HIF1α-dependent glycolysis promotes macrophage functional activities in protecting against bacterial and fungal infection. Sci Rep. (2018) 8:3603. doi: 10.1038/s41598-018-22039-9, PMID: 29483608 PMC5827022

[35] Jing X Du T Chen K Guo J Xiang W Yao X . Icariin protects against iron overload-induced bone loss via suppressing oxidative stress. J Cell Physiol. (2019) 234:10123–37. doi: 10.1002/jcp.27678, PMID: 30387158

[36] Zhang J Zhang L Yao G Zhao H Wu S . NRF2 is essential for iron-overload stimulated osteoclast differentiation through regulation of redox and iron homeostasis. Cell Biol toxicology. (2023) 39:3305–21. doi: 10.1007/s10565-023-09834-5, PMID: 37855941

[37] Zhang M Liu J Guo W Liu X Liu S Yin H . Icariin regulates systemic iron metabolism by increasing hepatic hepcidin expression through Stat3 and Smad1/5/8 signaling. Int J Mol Med. (2016) 37:1379–88. doi: 10.3892/ijmm.2016.2545, PMID: 27035325

[38] Si Y Li Y Gu K Yin H Ma Y . Icariin ameliorates osteoporosis in ovariectomized rats by targeting Cullin 3/Nrf2/OH pathway for osteoclast inhibition. Biomedicine pharmacotherapy = Biomedecine pharmacotherapie. (2024) 173:116422. doi: 10.1016/j.biopha.2024.116422, PMID: 38471268

[39] Yuan H Qiu Y Mei Z Liu J Wang L Zhang K . Cancer stem cells and tumor-associated macrophages: Interactions and therapeutic opportunities. Cancer letters. (2025) 624:217737. doi: 10.1016/j.canlet.2025.217737, PMID: 40274063

[40] Wang M Li C Liu Y Jin Y Yu Y Tan X . The effect of macrophages and their exosomes in ischemic heart disease. Front Immunol. (2024) 15:1402468. doi: 10.3389/fimmu.2024.1402468, PMID: 38799471 PMC11116575

[41] Zhang Y Zhang M Li M Miao M Shou D Tong P . Icariin-enhanced osteoclast-derived exosomes promote repair of infected bone defects by regulating osteoclast and osteoblast communication. Int J nanomedicine. (2024) 19:12389–407. doi: 10.2147/IJN.S483621, PMID: 39588260 PMC11587802

[42] Xu Q Chen G Liu X Dai M Zhang B . Icariin inhibits RANKL-induced osteoclastogenesis via modulation of the NF-κB and MAPK signaling pathways. Biochem Biophys Res Commun. (2019) 508:902–6. doi: 10.1016/j.bbrc.2018.11.201, PMID: 30538045

[43] Yang S Zhang X Liao X Ding Y Gan J . Icariin regulates RANKL-induced osteoclast differentiation via the ERα/c-Src/RANK signaling. Biomed materials (Bristol England). (2024) 19:025049. doi: 10.1088/1748-605X/ad2554, PMID: 38415738

[44] Xie Y Sun W Yan F Liu H Deng Z Cai L . Icariin-loaded porous scaffolds for bone regeneration through the regulation of the coupling process of osteogenesis and osteoclastic activity. Int J nanomedicine. (2019) 14:6019–33. doi: 10.2147/IJN.S203859, PMID: 31534334 PMC6682326

[45] Liu H Jiao Y Forouzanfar T Wu G Guo R Lin H . High-strength double-network silk fibroin based hydrogel loaded with Icariin and BMSCs to inhibit osteoclasts and promote osteogenic differentiation to enhance bone repair. Biomaterials advances. (2024) 160:213856. doi: 10.1016/j.bioadv.2024.213856, PMID: 38640877

[46] Tan J Chen Z Xu Z Huang Y Qin L Long Y . Small intestine submucosa decorated 3D printed scaffold accelerated diabetic bone regeneration by ameliorating the microenvironment. J materials Chem B. (2024) 12:9375–89. doi: 10.1039/D4TB00772G, PMID: 39189426

[47] Lazarov T Juarez-Carreño S Cox N Geissmann F . Physiology and diseases of tissue-resident macrophages. Nature. (2023) 618:698–707. doi: 10.1038/s41586-023-06002-x, PMID: 37344646 PMC10649266

[48] Hegarty LM Jones GR Bain CC . Macrophages in intestinal homeostasis and inflammatory bowel disease. Nat Rev Gastroenterol hepatol. (2023) 20:538–53. doi: 10.1038/s41575-023-00769-0, PMID: 37069320

[49] Yu Y Kim SM Park K Kim HJ Kim JG Kim SE . Therapeutic nanodiamonds containing icariin ameliorate the progression of osteoarthritis in rats. Int J Mol Sci. (2023) 24:15977. doi: 10.3390/ijms242115977, PMID: 37958960 PMC10647515

[50] Xu S Zhao S Jian Y Shao X Han D Zhang F . Icariin-loaded hydrogel with concurrent chondrogenesis and anti-inflammatory properties for promoting cartilage regeneration in a large animal model. Front Cell Dev Biol. (2022) 10:1011260. doi: 10.3389/fcell.2022.1011260, PMID: 36506090 PMC9730024

[51] Jiang C Somavarapu S . Trehalose-stabilized micelle-in-microparticles of icariin targeting IL-4 pathway in chronic obstructive pulmonary disease. Eur J Pharm sciences: Off J Eur Fed Pharm Sci. (2025) 212:107223. doi: 10.1016/j.ejps.2025.107223, PMID: 40754011

[52] Fu J Wang X . Improvement of RSV-induced asthma in mice: A study based on icariin-mediated PD-1. Front bioscience (Landmark edition). (2025) 30:26061. doi: 10.31083/FBL26061, PMID: 40152372

[53] Li J Zhao P Tian Y Li K Zhang L Guan Q . The anti-inflammatory effect of a combination of five compounds from five chinese herbal medicines used in the treatment of COPD. Front Pharmacol. (2021) 12:709702. doi: 10.3389/fphar.2021.709702, PMID: 34776947 PMC8578279

[54] Cong H Zhang M Chang H Du L Zhang X Yin L . Icariin ameliorates the progression of experimental autoimmune encephalomyelitis by down-regulating the major inflammatory signal pathways in a mouse relapse-remission model of multiple sclerosis. Eur J Pharmacol. (2020) 885:173523. doi: 10.1016/j.ejphar.2020.173523, PMID: 32871176

[55] Su B Ye H You X Ni H Chen X Li L . Icariin alleviates murine lupus nephritis via inhibiting NF-κB activation pathway and NLRP3 inflammasome. Life Sci. (2018) 208:26–32. doi: 10.1016/j.lfs.2018.07.009, PMID: 30146016

[56] Yang H Yan L Qian P Duan H Wu J Li B . Icariin inhibits foam cell formation by down-regulating the expression of CD36 and up-regulating the expression of SR-BI. J Cell Biochem. (2015) 116:580–8. doi: 10.1002/jcb.25009, PMID: 25389062

[57] Ma Y Yang H Huang J . Icariin ameliorates dexamethasone−induced bone deterioration in an experimental mouse model via activation of microRNA−186 inhibition of cathepsin K. Mol Med Rep. (2018) 17:1633–41. doi: 10.3892/mmr.2017.8065, PMID: 29257214 PMC5780104

[58] Zhou L Poon CC Wong KY Cao S Dong X Zhang Y . Icariin ameliorates estrogen-deficiency induced bone loss by enhancing IGF-I signaling via its crosstalk with non-genomic ERα signaling. Phytomedicine: Int J phytotherapy phytopharmacology. (2021) 82:153413. doi: 10.1016/j.phymed.2020.153413, PMID: 33339654

[59] Luo M Lin X Wang N Xie Q Zeng S Li S . Icariin delivery system based on covalent organic framework materials: dual effects of immune modulation and osteogenesis promotion. RSC advances. (2025) 15:23783–800. doi: 10.1039/D5RA01207D, PMID: 40630699 PMC12236428

[60] Elhakim A Kim S Shin SJ Jung HS Kim E . Effect of icariin surface treatment on the resorption of denuded roots after replantation in rat. Int endodontic J. (2023) 56:1550–8. doi: 10.1111/iej.13980, PMID: 37787769

[61] Guangtao F Zhenkang W Zhantao D Mengyuan L Qingtian L Yuanchen M . Icariin alleviates wear particle-induced periprosthetic osteolysis via down-regulation of the estrogen receptor α-mediated NF-κB signaling pathway in macrophages. Front Pharmacol. (2021) 12:746391. doi: 10.3389/fphar.2021.746391, PMID: 34803690 PMC8595199

[62] Wang J Tao Y Ping Z Zhang W Hu X Wang Y . Icariin attenuates titanium-particle inhibition of bone formation by activating the Wnt/β-catenin signaling pathway *in vivo* and *in vitro*. Sci Rep. (2016) 6:23827. doi: 10.1038/srep23827, PMID: 27029606 PMC4814911

[63] Xue L Jiang Y Han T Zhang N Qin L Xin H . Comparative proteomic and metabolomic analysis reveal the antiosteoporotic molecular mechanism of icariin from Epimedium brevicornu maxim. J ethnopharmacol. (2016) 192:370–81. doi: 10.1016/j.jep.2016.07.037, PMID: 27422162

[64] Zheng X Li D Li J Wang B Zhang L Yuan X . Optimization of the process for purifying icariin from Herba Epimedii by macroporous resin and the regulatory role of icariin in the tumor immune microenvironment. Biomedicine pharmacotherapy = Biomedecine pharmacotherapie. (2019) 118:109275. doi: 10.1016/j.biopha.2019.109275, PMID: 31382128

[65] Zhang X Kang Z Li Q Zhang J Cheng S Chang H . Antigen-adjuvant effects of icariin in enhancing tumor-specific immunity in mastocytoma-bearing DBA/2J mice. Biomedicine pharmacotherapy = Biomedecine pharmacotherapie. (2018) 99:810–6. doi: 10.1016/j.biopha.2018.01.139, PMID: 29710479

[66] Dongye Z Wu X Wen Y Ding X Wang C Zhao T . Icaritin and intratumoral injection of CpG treatment synergistically promote T cell infiltration and antitumor immune response in mice. Int immunopharmacol. (2022) 111:109093. doi: 10.1016/j.intimp.2022.109093, PMID: 35930912

[67] Reyes-Hernández OD Figueroa-González G Quintas-Granados LI Hernández-Parra H Peña-Corona SI Cortés H . New insights into the anticancer therapeutic potential of icaritin and its synthetic derivatives. Drug Dev Res. (2024) 85:e22175. doi: 10.1002/ddr.22175, PMID: 38567708

[68] Ban JQ Ao LH He X Zhao H Li J . Advances in macrophage-myofibroblast transformation in fibrotic diseases. Front Immunol. (2024) 15:1461919. doi: 10.3389/fimmu.2024.1461919, PMID: 39445007 PMC11496091

[69] Huang Y Wu F Zhan T Cao X Bao W Feng G . Icariin rescues immune dysfunction in sepsis through RSK2-driven YAP-cGAS-IFN-β signaling activation. Phytomedicine: Int J phytotherapy phytopharmacology. (2025) 149:157524. doi: 10.1016/j.phymed.2025.157524, PMID: 41240541

[70] Desai TD Wen YT Daddam JR Cheng F Chen CC Pan CL . Long term therapeutic effects of icariin-loaded PLGA microspheres in an experimental model of optic nerve ischemia via modulation of CEBP-β/G-CSF/noncanonical NF-κB axis. Bioengineering Trans Med. (2022) 7:e10289. doi: 10.1002/btm2.10289, PMID: 35600664 PMC9115698

[71] Darwish SF Elbadry AMM Elbokhomy AS Salama GA Salama RM . The dual face of microglia (M1/M2) as a potential target in the protective effect of nutraceuticals against neurodegenerative diseases. Front aging. (2023) 4:1231706. doi: 10.3389/fragi.2023.1231706, PMID: 37744008 PMC10513083

[72] Khodaei A Nawaz Q Zhu Z Amin Yavari S Weinans H Boccaccini AR . Biomolecule and ion releasing mesoporous nanoparticles: nonconvergent osteogenic and osteo-immunogenic performance. ACS Appl materials interfaces. (2024) 16:67491–503. doi: 10.1021/acsami.4c17540, PMID: 39576881 PMC11647896

[73] Chai H Sang S Luo Y He R Yuan X Zhang X . Icariin-loaded sulfonated polyetheretherketone with osteogenesis promotion and osteoclastogenesis inhibition properties via immunomodulation for advanced osseointegration. J materials Chem B. (2022) 10:3531–40. doi: 10.1039/D1TB02802B, PMID: 35416810

[74] Negrescu AM Mitran V Draghicescu W Popescu S Pirvu C Ionascu I . TiO(2) nanotubes functionalized with icariin for an attenuated *in vitro* immune response and improved *in vivo* osseointegration. J Funct biomaterials. (2022) 13:43. doi: 10.3390/jfb13020043, PMID: 35466225 PMC9036299

[75] Zhao F Chen F Song T Tian L Guo H Li D . One-step strategy for fabricating icariin-encapsulated biomimetic Scaffold: Orchestrating immune, angiogenic, and osteogenic cascade for enhanced bone regeneration. Bioactive materials. (2025) 52:271–86. doi: 10.1016/j.bioactmat.2025.06.001, PMID: 40547322 PMC12182316

[76] Qin SK Li Q Ming Xu J Liang J Cheng Y Fan Y . Icaritin-induced immunomodulatory efficacy in advanced hepatitis B virus-related hepatocellular carcinoma: Immunodynamic biomarkers and overall survival. Cancer science. (2020) 111:4218–31. doi: 10.1111/cas.14641, PMID: 32889778 PMC7648021

