# Supplementary material for: The multifaceted regulatory effect of icariin on macrophages: a mini-review
Source: Front Immunol. 2026 Jan 21;17:1765533. doi: 10.3389/fimmu.2026.1765533 (PMC12867808; doi:10.3389/fimmu.2026.1765533)
Supplement: Supplementary file 1 [file DataSheet1.pdf]

Supplementary materials

The multifaceted regulatory effect of icariin on macrophages: a mini-review

Juan Gao [1], Jin-Hong Gao [1], Yan-Fen Zhang [1], Da Gao [1], Ya-Peng Zhang [1,*]

[1] Department of Hematology, The Affiliated Hospital of Inner Mongolia Medical University, Hohhot 010030, China.

*Correspondence to:

Ya-Peng Zhang at 20122017@immu.edu.cn. Department of Hematology, The Affiliated Hospital of Inner Mongolia Medical University; Hohhot 010030, China. Tel.: +86-0471-3451083; fax: +86-0471-6965931.

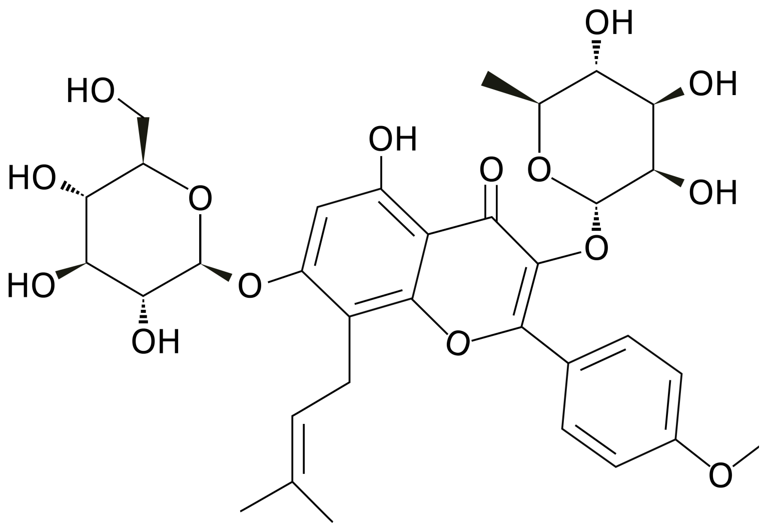

Supplemental Figure 1 The chemical structural formula of icariin

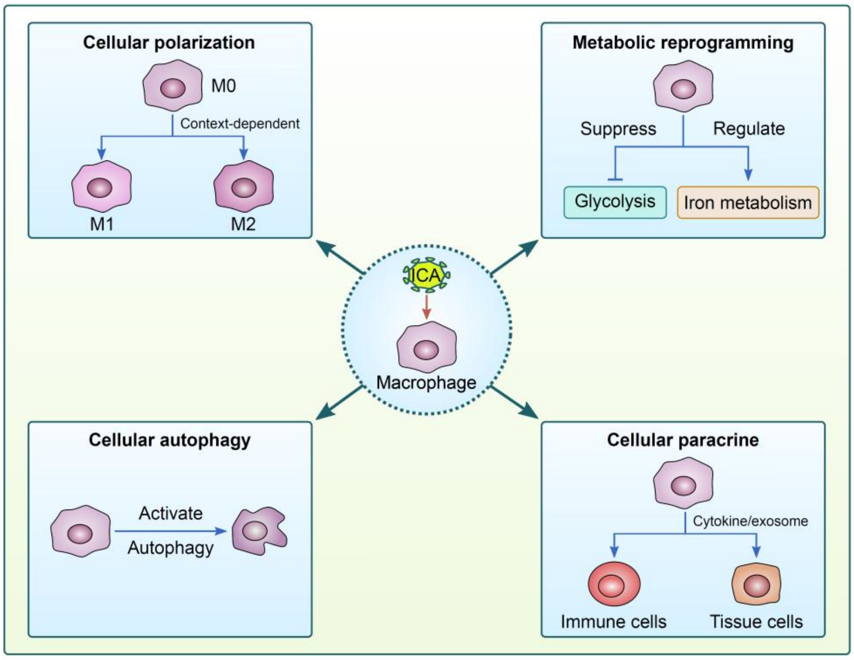

Supplemental Figure 2 The modulatory effect of ICA on macrophages

Icariin (ICA) regulates the polarization of macrophages into M1 or M2 subtypes in various microenvironments. It also modulates the metabolic reprogramming of macrophages to regulate glycolysis and iron metabolism. ICA can activate macrophage autophagy to exert anti-aging and anti-fibrotic effects. Moreover, it can modulate the function of other immune cells and tissue cells by secreting cytokines and exosomes.

Supplemental Table 1 Comparison of different macrophage-targeted delivery systems for ICA

	DLE	Release profiles	Targeting capability or effects on macrophages
**Exosome-based systems**			
Adipose-derived stem cell exosome (1)	92%	NA	• Increasing uptake by M1 macrophages<br>• Increasing inhibitory effect on M1 macrophage polarization<br>• Enhancing accumulation in inflammatory sites
**Hydrogel-based systems**			
Polyethylene glycol hydrogel (2)	NA	Nearly 70% release at 48 hours	• Promoting M2 macrophage polarization
hydroxybutyl chitosan hydrogel (3)	85%	Nearly 85% release at 16 days	• Promoting M2 macrophage polarization
Chitosan hydrogel (4)	NA	Nearly 100% release at 6 weeks	• Tendency of increasing inhibitory effect on macrophage function
**Nanoparticle-based systems**			
Nanodiamond nanoparticles (5)	79%	More than 96% release at 28 days	•Potentiating suppression of inflammation mediated by macrophages
Covalent organic framework nanoparticles (6)	33%	Nearly 70% release at 14 days	• Promoting M2 macrophage polarization<br>• Enhancing anti-inflammatory responses
Bioactive glass nanoparticles (7)	NA	Maximum release at 14 days	• Promoting M2 macrophage polarization
**Scaffold-based systems**			
PLGA scaffold (8)	NA	More than 80% release at 15 days	• Promoting M2 macrophage polarization
Sulfonated polyetheretherketone scaffold (9)	NA	Sustained release for 28 days	• Promoting M2 macrophage polarization
TiO2 Nanotubes scaffold (10)	NA	Sustained release for 14 days	•Potentiating suppression of inflammation mediated by macrophages
**Micelle-in-microparticle systems**			
Trehalose-stabilized microparticles (11)	91%	Nearly 93% release during the observation period	• Increasing uptake by macrophages<br>• Suppressing M2 macrophage polarization
PLGA biomimetic microparticles (12)	90%	Nearly 20% release at 13 days	• Promoting M2 macrophage polarization

DLE: drug-loading efficacy; PLGA: poly(lactic-co-glycolic acid); NA: not available.

References

1. Yan Q, Liu H, Sun S, Yang Y, Fan D, Yang Y, et al. Adipose-derived stem cell exosomes loaded with icariin alleviates rheumatoid arthritis by modulating macrophage polarization in rats. Journal of nanobiotechnology. 2024; 22(1): 423.

2. Teng YY, Zou ML, Liu SY, Jia Y, Zhang KW, Yuan ZD, et al. Dual-Action Icariin-Containing Thermosensitive Hydrogel for Wound Macrophage Polarization and Hair-Follicle Neogenesis. Frontiers in bioengineering and biotechnology. 2022; 10: 902894.

3. Chen J, Guan X, Chen L, Zheng B, Li F, Fang C, et al. Customized Hydrogel System for the Spatiotemporal Sequential Treatment of Periodontitis Propelled by ZEB1. Advanced science (Weinheim, Baden-Wurttemberg, Germany). 2025; 12(26): e2503338.

4. Xu S, Zhao S, Jian Y, Shao X, Han D, Zhang F, et al. Icariin-loaded hydrogel with concurrent chondrogenesis and anti-inflammatory properties for promoting cartilage regeneration in a large animal model. Frontiers in cell and developmental biology. 2022; 10: 1011260.

5. Yu Y, Kim SM, Park K, Kim HJ, Kim JG, Kim SE. Therapeutic Nanodiamonds Containing Icariin Ameliorate the Progression of Osteoarthritis in Rats. International journal of molecular sciences. 2023; 24(21): 15977.

6. Luo M, Lin X, Wang N, Xie Q, Zeng S, Li S, et al. Icariin delivery system based on covalent organic framework materials: dual effects of immune modulation and osteogenesis promotion. RSC advances. 2025; 15(29): 23783-800.

7. Khodaei A, Nawaz Q, Zhu Z, Amin Yavari S, Weinans H, Boccaccini AR. Biomolecule and Ion Releasing Mesoporous Nanoparticles: Nonconvergent Osteogenic and Osteo-immunogenic Performance. ACS applied materials & interfaces. 2024; 16(49): 67491-503.

8. Tan J, Chen Z, Xu Z, Huang Y, Qin L, Long Y, et al. Small intestine submucosa decorated 3D printed scaffold accelerated diabetic bone regeneration by ameliorating the microenvironment. Journal of materials chemistry B. 2024; 12(37): 9375-89.

9. Chai H, Sang S, Luo Y, He R, Yuan X, Zhang X. Icariin-loaded sulfonated polyetheretherketone with osteogenesis promotion and osteoclastogenesis inhibition properties via immunomodulation for advanced osseointegration. Journal of materials chemistry B. 2022; 10(18): 3531-40.

10. Negrescu AM, Mitran V, Draghicescu W, Popescu S, Pirvu C, Ionascu I, et al. TiO(2) Nanotubes Functionalized with Icariin for an Attenuated In Vitro Immune Response and Improved In Vivo Osseointegration. Journal of functional biomaterials. 2022; 13(2): 43.
11. Jiang C, Somavarapu S. Trehalose-stabilized micelle-in-microparticles of icariin targeting IL-4 pathway in chronic obstructive pulmonary disease. European journal of pharmaceutical sciences : official journal of the European Federation for Pharmaceutical Sciences. 2025;212:107223.
12. Zhao F, Chen F, Song T, Tian L, Guo H, Li D, et al. One-step strategy for fabricating icariin-encapsulated biomimetic Scaffold: Orchestrating immune, angiogenic, and osteogenic cascade for enhanced bone regeneration. Bioactive materials. 2025;52:271-86.
